# Mammogram image quality as a potential contributor to disparities in breast cancer stage at diagnosis: an observational study

**DOI:** 10.1186/1471-2407-13-208

**Published:** 2013-04-26

**Authors:** Garth H Rauscher, Emily F Conant, Jenna A Khan, Michael L Berbaum

**Affiliations:** 1 School of Public Health, Division of Epidemiology and Biostatistics, University of Illinois at Chicago, M/C 923, Chicago, IL 60612, USA; 2 Department of Radiology/Breast Imaging, University of Pennsylvania, 3400 Spruce Street, Philadelphia, PA 19104, USA; 3 Institute for Health Research and Policy University of Illinois at Chicago, M/C 275, 1747 West Roosevelt Road, Chicago, IL 60608, USA

**Keywords:** Breast cancer, Disparities, Screening, Mammography, Socioeconomic status

## Abstract

**Background:**

In an ongoing study of racial/ethnic disparities in breast cancer stage at diagnosis, we consented patients to allow us to review their mammogram images, in order to examine the potential role of mammogram image quality on this disparity.

**Methods:**

In a population-based study of urban breast cancer patients, a single breast imaging specialist (EC) performed a blinded review of the index mammogram that prompted diagnostic follow-up, as well as recent prior mammograms performed approximately one or two years prior to the index mammogram. Seven indicators of image quality were assessed on a five-point Likert scale, where 4 and 5 represented good and excellent quality. These included 3 technologist-associated image quality (TAIQ) indicators (positioning, compression, sharpness), and 4 machine associated image quality (MAIQ) indicators (contrast, exposure, noise and artifacts). Results are based on 494 images examined for 268 patients, including 225 prior images.

**Results:**

Whereas MAIQ was generally high, TAIQ was more variable. In multivariable models of sociodemographic predictors of TAIQ, less income was associated with lower TAIQ (p < 0.05). Among prior mammograms, lower TAIQ was subsequently associated with later stage at diagnosis, even after adjusting for multiple patient and practice factors (OR = 0.80, 95% CI: 0.65, 0.99).

**Conclusions:**

Considerable gains could be made in terms of increasing image quality through better positioning, compression and sharpness, gains that could impact subsequent stage at diagnosis.

## Background

In the United States there is evidence that non-Hispanic (nH) Black women are more likely to die from breast cancer compared to their nH White counterparts, despite having a lower incidence of the disease. This mortality disparity is especially high in Chicago, where most recent available data suggests that nH Black women die from breast cancer at a two thirds higher rate than nH Whites [1]. Despite current controversies regarding the timing and frequency of screening with mammography [2-6], it is generally recognized as effective in reducing morbidity and mortality from breast cancer [7,8]. Despite reporting similar mammography utilization [9], Black and Hispanic women continue to be diagnosed at a later stage of breast cancer compared to Whites [10] and this later stage is at least partly responsible for the greater breast cancer mortality experienced by Black women in the United States as compared to Whites.

Prior data from Chicago suggest that nH Black and Hispanic women were less likely than nH Whites to obtain screening mammography at facilities with characteristics suggesting high quality screening, which include academic facilities, facilities that relied on breast imaging specialists, and facilities that offered digital mammography [11]. These apparent disparities in the distribution of mammography practice characteristics might result in a disparity in the quality of the process of mammography screening and diagnostic follow-up. Racial/ethnic or socioeconomic disparities in quality of mammogram images, radiologic interpretation of mammograms, or timeliness of diagnostic follow-up and resolution of an abnormal mammogram might be mediated by mammography practice characteristics. The goals of the present analysis were to (1) examine whether better image quality was associated with earlier breast cancer stage at diagnosis, and (2) examine whether there existed disparities in image quality by race/ethnicity or socioeconomic status.

Our hypothesis was that racial and ethnic minorities and women of lower socioeconomic status (less education and income, and lacking private health insurance) would tend to be screened at lower resource facilities. These might include facilities not situated in academic medical centers, that relied to a lesser extent on radiologist and technologist specialists, and that tended to use analog as opposed to digital mammography. Digital mammograms may tend to be of higher quality than analog images [12].

This unequal distribution of mammography practice characteristics might translate into lower image quality for these women. We conceptualized image quality as having two components, one related to the skill of the technologist and one related primarily to mammography machine calibration. Lower image quality has been associated with interval breast cancer, i.e., breast cancer presenting through symptoms despite a recent normal-appearing screening mammogram [13]. Symptomatic breast cancer is considerably more likely to be later stage than screen-detected breast cancer [14]; thus, image quality might be associated with stage at diagnosis and might help to explain disparities in stage at diagnosis.

## Materials and methods

### Sample and procedure

Patients for this study were recruited from the parent study, “Breast Cancer Care in Chicago”, details of which have been previously published [15]. Briefly, female patients were eligible if they were diagnosed between March 1, 2005 and February 31, 2008, diagnosed between 30 and 79 years of age, resided in Chicago, had a first primary in situ or invasive breast cancer, and self-identified as either non-Hispanic White, non-Hispanic Black or Hispanic. All diagnosing facilities in the greater Chicago area (N = 56) were visited monthly by certified tumor registrars employed by the Illinois State Cancer Registry (ISCR) and all eligible newly diagnosed cases were ascertained. Participants completed a 90-minute interview that was administered either in English or Spanish as appropriate using computer-assisted personal interview procedures. The final interview response rate was 56% representing 989 completed interviews among eligible patients (397 nH White, 411 nH Black, 181 Hispanic, response rates 51%, 59% and 66%, respectively) [16]. Upon completion of the interview, patients were asked to provide consent to allow abstraction of their medical records for information pertaining to their breast cancer diagnosis, and asked to allow the study to obtain original breast screening and diagnostic images for the mammography review substudy. Both the main study and mammogram review substudy were reviewed and approved by the University of Illinois at Chicago Office for the Protection of Research Subjects.

### Mammogram review substudy

Patients reporting either initial awareness of their breast cancer through screening mammography or initial awareness through symptoms despite a prior mammogram within 2 years of detection were eligible for this substudy (N = 597). Of these, 369 (62%) consented to a review of their mammogram and other breast images involved in their screening and diagnosis. Original mammograms, diagnostic follow-up images and corresponding reports were requested from screening and diagnostic facilities. Often, multiple facilities were involved for a single patient. In all, we received 494 mammograms performed on 268 patients. Approximately 90% of mammograms were bilateral, standard four view mammograms, while the remainders were unilateral mammograms.

A single breast imaging specialist (EC) performed a blinded review of mammograms (blinded to the original interpretation and all other subsequent screening and diagnostic mammograms and results). All reviews were blinded to patient age, race/ethnicity and other sociodemographic characteristics. Seven indicators of image quality were assessed: positioning, compression, sharpness, contrast, exposure, noise and artifacts [13,17]. Each was scored on a five-point Likert scale, where 4 and 5 represented good and excellent quality, respectively, while 1 represented poor quality. The 273 participants were less likely than eligible nonparticipants to be minority (46% vs. 64%, p < 0.0005) and more likely to report symptomatic discovery (43% vs. 31%, p = 0.002), but were similar on other characteristics.

### Analysis variables

#### Variables for image quality

We defined a continuous measure of image quality that was a simple sum of the 7 indicators, with a theoretical range of 7 (lowest quality) to 35 (highest quality). Results of analyses using this variable were similar to results using the binary version described next, and therefore these results are not presented. We defined a separate binary variable to indicate higher image quality as those images that received a score of at least 4 (very good) on all seven indicators. Positioning, compression, and sharpness are affected by the patient-technologist interaction and the skill of the technologist, whereas contrast, noise, exposure, and artifacts are primarily a function of mammography machine calibration. Therefore, we defined two additional measures of image quality. Higher technologist-associated image quality (TAIQ) was defined as receiving a score of at least 4 (very good) on positioning, compression, and sharpness, and a variable summing these three measures was defined. Higher machine-associated image quality (MAIQ) was defined as receiving a score of at least 4 (very good) on contrast, noise, exposure, and artifacts, and a variable summing these four measures was defined.

### Measures of race/ethnicity and socioeconomic disadvantage

Race and ethnicity were self-reported at interview. Ethnicity was defined as Hispanic if the patient self-identified as Hispanic, or reported a Latin American country of origin for herself or for both of her biological parents. Race and ethnicity were used to categorize patients as non-Hispanic White, non-Hispanic Black or Hispanic. Socioeconomic disadvantage was defined from self-reported annual household income, educational attainment, and health insurance status. Income was reported at interview in categories of less than $10,000, $20,000, $30,000, $40,000, $50,000, $75,000, $100,000, $150,000, $200,000, and greater than $200,000. Annual household income analyzed as an ordinal variable and also categorized for some analyses as not exceeding versus exceeding $30,000. Formal education was reported in years, and was analyzed both as an ordinal variable and categorized as not exceeding a high-school degree versus having some post-secondary education. Health insurance status was categorized as lacking private health insurance versus having any private health insurance. Patients with Medigap or similar supplementary private health insurance were defined as privately insured.

### Mammography practice characteristics

Individual mammograms were defined with respect to image type as either digital or analog (film screen). Although image type is an individual attribute of the mammogram itself, it indicates something about the availability of digital mammography and therefore we group it here with practice characteristics. All mammography facilities included in these analyses were accredited by the Mammography Quality Standards Act (MQSA). Mammography practices were grouped by facility type into: (a) public, (b) private non-academic, (c) private with an academic-affiliation, or (d) private university-based hospital or medical center. For some analyses we dichotomized this variable to indicate whether a facility was located within an academic hospital or medical center. Facility data on the numbers and types of mammography technicians and radiologists were available from a prior mammography facility survey of Chicago performed during the study period [11]. Facilities reported the number of general radiologists and breast imaging specialists interpreting mammographic studies. A breast imaging specialist was defined as a radiologist who dedicated at least 75% of his/her working time on breast imaging, regardless of fellowship training. We defined a variable describing each facility’s reliance on breast imaging specialists as none, mixed, or sole reliance on specialists. We defined an analogous variable to categorize facilities by the extent of their reliance on dedicated mammography technologists (mixed vs. sole).

### Clinical variables

Mode of breast cancer detection was defined as asymptomatic if the patient reported initial awareness of the breast cancer through mammography or other breast imaging in the absence of any symptoms, otherwise mode of detection was defined as symptomatic. Stage at diagnosis was categorized using the AJCC categories of 0, 1, 2, 3, and 4 ( http://www.cancerstaging.org/).

### Statistical analyses

Statistical analyses were conducted using SAS version 9 (SAS Institute, Cary, NC) and Stata version 11 (Statacorp, College Station, Texas). We tabulated the observed distribution for each of the seven image quality indicators and estimated polychoric correlations [18]. The percentage of higher image quality mammograms was tabulated against patient characteristics and mammography practice characteristics, and was repeated for higher technologist-associated image quality and higher machine-associated image quality. Due to the greater variability of TA image quality, only these associations are presented. P-values were estimated in univariable logistic regressions using the Huber-White sandwich estimator to adjust standard errors for clustering of images within patients.

### Multivariable logistic regression of higher image quality

Due to the greater variability of TA image quality, we focused on these image quality indicators in the analyses that follow. We conducted multivariable logistic regression models in order to estimate associations with higher TA image quality while using the Huber-White sandwich estimator to adjust standard errors for clustering of images within patients. The first model (baseline model) included terms for age and age squared. Next, we added variables for race/ethnicity, income, education and private insurance status together. Through backwards elimination procedures we removed those variables with a p-value >0.10 via Wald tests.

### Image quality indicators as predictors of stage at diagnosis

After excluding the index films and using data from only the prior films (performed prior to any symptoms or diagnosis), we conducted multivariable ordinal logistic regression to estimate associations for higher TA image quality with breast cancer stage at diagnosis. Each image quality indicator was modeled separately while adjusting for age, education, income, private health insurance status, academic vs. other facility type and image type (analog vs. digital). We estimated odds ratios from ordinal logistic regression models with robust standard errors.

## Results

### Image quality

Results are based on 494 images examined for 268 patients. Very good or excellent scores were less frequent for positioning, compression and sharpness (61, 66, and 68%) than for contrast, exposure, artifacts and noise (86, 89, 90 and 96%, respectively) (Table  1). As anticipated, polychoric correlations between the seven mammography quality indicators were higher within technologist-associated indicators (mean 0.86, range 0.79-0.94), and higher within machine-associated indicators (mean 0.84, range 0.77-0.93), than between technologist-associated and machine-associated indicators (mean 0.63, range 0.51-0.72) (Table  2).

**Table 1:** Distribution of image quality indicators (N = 494 images)

	**Poor**	**Moderate**	**Good**	**Very good**	**Excellent**
	**%**	**%**	**%**	**%**	**%**
Technologist-Associated					
Positioning	1	7	32	54	7
Compression	0	4	29	62	4
Sharpness	0	4	27	62	6
Machine-Associated					
Contrast	1	1	12	75	11
Exposure	0	1	9	78	11
Artifacts	1	2	8	77	13
Noise	0	0	3	87	9

**Table 2:** Polychoric correlations between the seven mammography quality indicators

	**Technologist-associated**			**Machine-associated**			
	**Positioning**	**Compression**	**Sharpness**	**Contrast**	**Noise**	**Exposure**	**Artifacts**
Technologist-associated							
Positioning	1						
Compression	0.84	1					
Sharpness	0.79	0.94	1				
Machine-associated							
Contrast	0.54	0.64	0.64	1			
Noise	0.58	0.67	0.72	0.86	1		
Exposure	0.51	0.62	0.69	0.93	0.85	1	
Artifacts	0.60	0.63	0.71	0.77	0.85	0.80	1

### Patient and practice characteristics predict lower image quality

Racial/ethnic and socioeconomic disadvantage were associated with lower TA image quality (Table  3). The percentage of films that received a score of 4 or 5 on all 3 TA indicators was greater for nH White than minority patients (57% vs. 49%, p = 0.13), greater for patients reporting higher vs. lower income (58% vs. 45%, p = 0.03), and greater for patients with more than a high-school education than for those with less education (58% vs. 46%, p = 0.04), but did not vary by private health insurance status (Table  3). Better image quality was considerably more likely for images performed at hospital-based, academic facilities than at other types of facilities. The extent to which facilities relied on full-time mammography technologists did not seem to be associated with image quality. On the other hand, better image quality was considerably more likely for images performed at facilities that relied solely on breast imaging specialists than at facilities that did not (Table  3). Digital mammograms were considerably more likely to be scored as high quality than analog images. Results were generally similar when we examined all 7 indicators as a group. There was little variation in machine-associated image quality indicators by racial/ethnicity or socioeconomic status (results not shown).

**Table 3:** Distribution of patient and practice characteristics with higher technologist-associated image quality

	**N**	**(%)**	**p-value**
Race/ethnicity			0.13
non-Hispanic White	268	57	
Black or Hispanic	221	49	
Annual household income			0.03
Higher (>$30,000)	320	58	
Lower (<$30,000)	152	45	
Educational attainment			0.04
More than high-school	326	58	
High-school degree or less	159	46	
Health insurance status			
Some private insurance	380	55	
No private insurance	109	50	
Facility type			<0.0001
Public	31	48	
Private, non-academic	294	44	
Academic (affiliate)	38	53	
Academic (hospital)	126	78	
Mammography interpretation			<0.0001
Sole reliance on generalists	111	40	
Mixed reliance	153	49	
Sole reliance on specialists	170	74	
Sole reliance on dedicated techs			
No	271	57	
Yes	163	55	
Type of mammogram			<0.0001
Analog	345	42	
Digital	144	82	

P-values >0.2 are suppressed. P-values calculated from logistic regression of image quality indicator against each characteristic and accounting for clustering of multiple images per patient.

### Multivariable models of higher image quality

We conducted multivariable logistic regression in order to examine the extent to which race/ethnicity and socioeconomic status were associated with image quality. When racial/ethnic and socioeconomic variables were modeled together in logistic regression models of higher image quality, only income was retained (p-value for income = 0.001) while race/ethnicity, health insurance status and education were not retained in the final model (Table  4). Results were very similar when modeling higher image quality based solely on technologist-associated indicators (p-value for income = 0.001), and again when modeling higher image quality based solely on machine-associated indicators (p-value for income = 0.03); in each instance, income and only income was retained (results not shown).

**Table 4:** Multivariable nested logistic regression models of higher image quality

	**Model 1**	**Model 2**	**Model 3**	**Model 4**
**Predictor**	**OR**	**OR**	**OR**	**OR**
Age (years)	1.01	1.01	1.01	1.01
Age*Age	1.00*	1.00*	1.00*	1.00*
Race/Ethnicity				
nH White				
nH Black	0.84			
Hispanic	1.13			
No private insurance	1.28	1.28		
Education (years)	1.07	1.07	1.05	
Income ($10,000 increments)	1.05*	1.06*	1.06*	1.07**
N	472	472	472	472
Log-Likelihood	−314.94	−315.48	−315.93	−317.03
LR Test (p-value)	---	0.58	0.34	0.14
AIC	645.88	642.95	641.87	642.05
BIC	679.14	667.9	662.65	658.68
Pseudo-R2	0.04	0.04	0.03	0.03

Legend: * p < .1; **p < .01; *** p < .001.

### TA image quality predicts stage at diagnosis

In order to examine the extent to which variation in image quality was associated with breast cancer stage at diagnosis, we conducted multivariable ordinal logistic regression models. Higher image quality across all seven indictors combined was inversely associated with breast cancer stage at diagnosis (OR = 0.91, 95% CI: 0.80, 1.03) (Table  5). Higher image quality for technologist-associated indicators was associated with earlier stage at diagnosis, whereas higher image quality for machine-associated indicators was generally not associated with stage at diagnosis (Table  5).

**Table 5:** Higher quality mammography imaging and breast cancer stage at diagnosis (N = 210 images prior to the index image with complete data on covariates)

	**OR (95% CI)**	**P-value**
All 7 image quality indicators^1^	0.91 (0.80, 1.03)	0.14
Technologist-associated		
Sum of all 3 indicators^1^	0.80 (0.65, 0.99)	0.04
Positioning	0.83 (0.49, 1.42)	
Compression	0.50 (0.29, 0.86)	0.01
Sharpness	0.54 (0.31, 0.92)	0.02
Machine-associated		
All 4 indicators^1^	0.96 (0.76, 1.22)	
Contrast	0.93 (0.50, 1.75)	
Exposure	1.03 (0.49, 2.15)	
Noise	0.53 (0.17, 1.61)	
Artifacts	0.98 (0.48, 1.99)	

Higher quality mammography imaging defined as a score of good or excellent for a given image quality indicator. ^1^ The number of indicators in the set that were scored as being of good or excellent quality. Odds ratios are from ordinal logistic regression models adjusted for age, race/ethnicity, income, education, private health insurance status, film type and facility type (academic medical center vs. other). P-values > 0.2 are suppressed.

## Discussion

In our population-based sample of urban breast cancer patients, lower technologist-associated image quality was associated with later breast cancer stage at diagnosis, and patients with lower income were less likely to obtain high quality mammography imaging. Our results suggest that differences in mammogram image quality may be contributing to socioeconomic disparities in stage at diagnosis.

Most of the variation in image quality was due to quality indicators that would tend to be associated with the skill of the technologist in working with the patient to ensure proper positioning, adequate compression, minimizing blur (maximizing sharpness), and performing the mammogram again if the quality of the image was suboptimal. Mammography technologists who spend most or all of their time performing mammograms may produce higher quality mammography images compared to technologists whose duties are split, although there is little if any literature on this topic. We anticipated that image quality would be better at facilities that relied solely on dedicated mammography technologists, but we did not find any evidence to support this expectation in the current study. We did find that mammogram images from academic institutions and from institutions relying on breast imaging specialists tended to be of higher quality. It may be that mammography technologists who work alongside breast imaging specialists have greater opportunities to expand their knowledge and expertise, or these settings may tend to hire more skilled technologists to begin with. A specialized, high volume breast radiologist may be better able to find cancers in images that are of lower quality than general radiologist or one that reads fewer mammograms. A tendency for lower quality imaging at non-academic facilities, in combination with less expertise available to read those images, may result in less early detection and later stage at diagnosis.

Prior studies have found that digital mammogram images are more likely to be judged as being higher quality compared to analog images [12]. In the present study, our expert consistently scored digital images as being of higher quality across all seven image quality indicators. Since we were unable to blind our expert as to whether an image being reviewed was analog or digital, it is conceivable that a bias or preference towards digital images may have resulted in an artificially higher image quality score for digital images, but this seems unlikely. Nonetheless, after controlling for type of mammogram in our analyses, lower income remained associated with lower quality imaging, and lower technologist-associated image quality was in turn associated with later stage diagnosis. Therefore, our results do not appear to be due to variation in the use of digital versus analog mammography across the study sample.

The Mammography Quality Standards Act (MQSA) was passed in 1992 in an attempt to improve the quality of breast cancer screening with mammography and provided basic standards that have to be met in order for a facility to be certified under the Act [19]. These included standards relevant to image quality, including mammography machine calibration and maintenance and qualifications of mammography technologists. However, mammogram images are only required to be reviewed under MQSA at least once every 3 years, and MQSA inspects only a small sample of images that are hand-picked in advance by the facility. Therefore, it could be a simple matter for a lower-performing institution to pass image quality inspection regardless of actual practice.

## Conclusions

We found that patients of higher socioeconomic status obtained higher quality images, and higher quality images were in turn associated with earlier stage at diagnosis. In particular, results suggest that considerable gains could be made in terms of increasing image quality through better positioning, compression and sharpness, which could translate into earlier stage at diagnosis for patients.

## Abbreviations

AJCC: American Joint Commission on Cancer; ISCR: Illinois State Cancer Registry; MQSA: Mammography Quality Standards Act; NC: North Carolina; nH: non-Hispanic; OR: Odds Ratio.

## Competing interests

The authors declare that they have no conflict of interest.

## Authors’ contributions

GR conceived of the study, and participated in its design and coordination and drafted the manuscript. EC performed the assessment of image quality that formed the basis for the analyses. JK participated in the statistical analysis. MB oversaw the statistical analysis. All authors participated in manuscript revisions and read and approved the final manuscript.

## Pre-publication history

The pre-publication history for this paper can be accessed here:

http://www.biomedcentral.com/1471-2407/13/208/prepub
